# Design of a Versatile pH-Responsive Hydrogel for Potential Oral Delivery of Gastric-Sensitive Bioactives

**DOI:** 10.3390/polym9100474

**Published:** 2017-09-27

**Authors:** Angus R. Hibbins, Pradeep Kumar, Yahya E. Choonara, Pierre P. D. Kondiah, Thashree Marimuthu, Lisa C. du Toit, Viness Pillay

**Affiliations:** Wits Advanced Drug Delivery Platform Research Unit, Department of Pharmacy and Pharmacology, Faculty of Health Sciences, School of Therapeutics Sciences, University of the Witwatersrand, Johannesburg, 7 York Road, Parktown 2193, South Africa; ahibbins@yahoo.com (A.R.H.); pradeep.kumar@wits.ac.za (P.K.); yahya.choonara@wits.ac.za (Y.E.C.); pierre.kondiah@wits.ac.za (P.P.D.K.); thashree.marimuthu@wits.ac.za (T.M.); lisa.dutoit@wits.ac.za (L.C.d.T.)

**Keywords:** acrylamide (Aam), methacrylic acid (MAA), stimuli-responsive drug delivery, pH-responsive hydrogel, free radical polymerization

## Abstract

A pH-responsive hydrogel system was prepared by free radical polymerization of acrylamide and methyl acrylic acid in the presence of *N*-*N*′-methylene bisacrylamide. Sodium bicarbonate was further applied as a blowing agent, which afforded a porous hydrogel structure. The hydrogel system achieved a constant super swelling rate within simulated intestinal buffer (~4%/min) and remained relatively static within simulated gastric buffer (~0.8%/min). The hydrogel system was able to achieve matrix resilience greater than 30% under a relatively high strain of 40%. In addition, the hydrogel system demonstrated significant swelling properties in response to simulated intestinal environmental over 24 h, with contrasting characteristics in simulated gastric buffer. The hydrogel demonstrated type IV isotherm porosity characteristics, with remarkable MRI and SEM variations in gastric and intestinal simulated fluids. Drug loading was observed to be greater than 98% using theophylline as a prototype drug, evaluating its controlled release kinetics over 24 h. The hydrogel exhibited substantial pH-responsive activity, which could be used as a versatile platform for targeted release of gastric-sensitive therapeutics to the small intestine.

## 1. Introduction

Hydrogels are simple yet unique and versatile in design and have therefore shown paramount application in the field of drug delivery [1,2,3]. The use of a pH-sensitive hydrogel that will undergo swelling when transiting through the gastrointestinal tract from an acidic (gastric environment) to a mildly basic (small intestinal environment) condition has been a significant focal point for many researchers [1,2,3,4,5,6]. The small intestine is considered the most desirable location within the gastrointestinal tract for the delivery of gastric-sensitive bioactives [7,8,9,10,11,12]. However, traditional hydrogels are limited by the speed at which they can swell within a respective fluid, being a limiting factor for effective use of stimuli-responsive hydrogels for drug delivery applications [13]. The design of a novel drug delivery device that utilizes a superporous pH responsive hydrogel as a chemicomechanical mechanism in order to release therapeutics within a site-specific location in the gastrointestinal tract is one approach to increasing the effectiveness of gastric-sensitive bioactives [14,15,16].

A superporous hydrogel (SPH) may also offer targeted delivery of sensitive bioactives within the gastrointestinal tract, provided external protection of the bioactive is maintained from the selected delivery system. The network structure found in SPH’s allows for large amounts of water to be retained within the network structure even when the hydrogel is removed from the aqueous environment [15]. A pH responsive hydrogel which would effectively swell in response to basic media is comprised of acidic monomers with functional groups of –OH, –COOH, –CONH_2_, and –SO_3_H [13]. The equilibrium degree of swelling is achieved slowly within the gastric environment. The slow degree of equilibrium could generate a positive pressure within a drug delivery device and prime the release of the bioactive within the small intestine. The equilibrium degree of swelling increases as the hydrogel is transitioned to basic conditions [14].

**Scheme 1:**
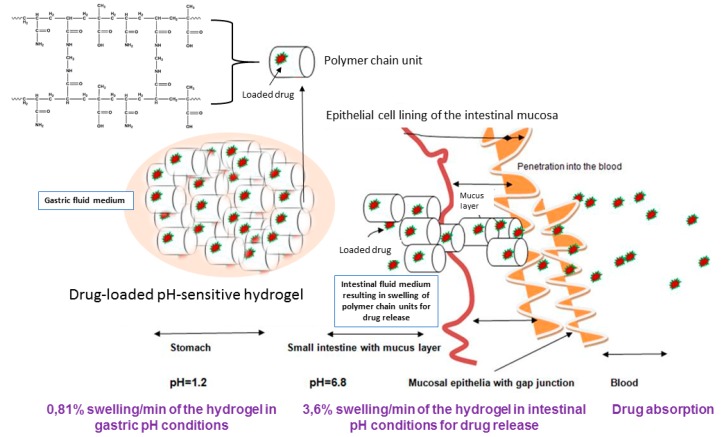
Mechanistic illustration of the pH-responsive hydrogel, swelling to a minimum degree in gastric pH and substantial swelling and release of drug in intestinal pH for absorption of the loaded drug in the small intestine.

In this research paper, we discuss the synthesis and evaluation of a pH-responsive hydrogel, designed to deliver sensitive bioactives via the oral route of administration, protecting its loaded bioactive in acidic medium and releasing the bioactive once the pH is adjusted to intestinal conditions. As seen in Scheme 1, the pH-responsive hydrogel allows significant protection of the sensitive bioactive, which is loaded in the polymeric matrix of the hydrogel, which can be attributed to minimum swelling capacity in acidic pH. Thus, due to the non-ionization of the functional groups in acidic medium, the hydrogel retains the loaded bioactive in the hydrogel matrix. The opposite is displayed in intestinal medium, where the acidic functional groups of the polymer are ionized, gradually swelling to a considerable degree, thus releasing the loaded bioactive concurrently.

The use of acrylamide and methacrylic acid to produce a pH-responsive hydrogel, designed for minimum swelling in gastric pH and maximum swelling and release of drugs in intestinal medium, was the defining characteristic of the delivery platform. With this conceptual objective, sensitive bioactives such as peptides and proteins can be well protected in the polymeric matrix of the hydrogel, and released intact once reaching the small intestine. In addition to the swelling properties of the pH-responsive hydrogel, in depth studies evaluating the physicomechanical properties in response to simulated gastric and intestinal fluid was also undertaken. In addition, the characterization of the superporous pH-responsive hydrogel with respect to simulated gastric and intestinal fluid is presented. The hydrogel was synthesized using acrylamide (Aam) and methacrylic acid (MAA). The monomers employed for synthesis, provided the swelling properties which are desired for this application, enabling the pH-responsiveness of the hydrogel to undergo highly controlled swelling within simulated intestinal conditions.

## 2. Materials and Methods

### 2.1. Materials

Acrylamide (≥98% pure, *M*_W_ 71.08 g/mol) was purchased from Fluka^®^ (Seelze, Germany), methacrylic acid (99% pure, 86.09 g/mol), tetramethylethylenediamine (99% pure, 116.20 g/mol), ammonium persulfate (98% pure, 228.20 g/mol), pluronic F-127 and *N*,*N*′-Methylenebis(acrylamide) (MBA) (99% pure, 154.17 g/mol) was purchased from Sigma-Aldrich^®^ (St. Louis, MO, USA).

### 2.2. Synthesis of the pH-Responsive Hydrogel

**Scheme 2:**
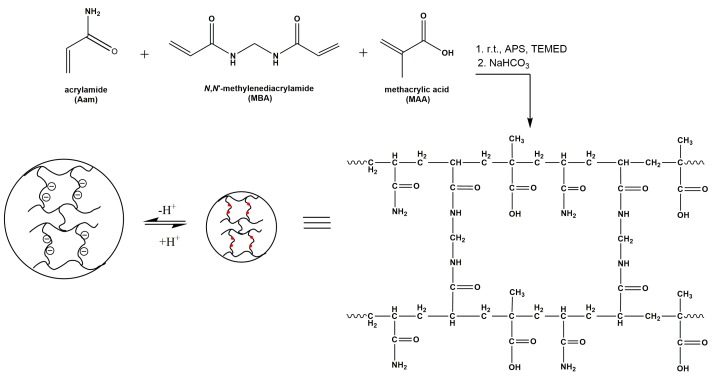
Free radical polymerization reaction of acrylamide (Aam) and methacrylic acid (MAA) as a pH-sensitive hydrogel oral drug delivery platform, illustrating the swelling and network shrinking of the copolymer in intestinal and gastric simulated medium respectively.

Variations of the hydrogel were synthesized via in situ free radical copolymerization of AaM and MAA as seen in Scheme 2. To an aqueous solution containing 50% *w*/*v* of AaM and MAA respectively, MBA (2.5% *w*/*v*), deionized water (30 mL) and Pluronic F-127 (10% *w*/*v*) were sequentially added, allowing the formulation to react under constant magnetic stirring of 3500 rpm. Thereafter APS (10% *w*/*v*) and TEMED (20% *w*/*v*) were added at room temperature before the addition of sodium bicarbonate (100 mg). The resulting solution was poured into cylindrical rod molds (inner diameter of 4.77 mm) and allowed to settle. The molds where placed in a glass beaker and incubated in a water bath at 37 °C (±0.5 °C) for 12 h. Once the purification process was completed, the in situ rods were then removed from the cylindrical molds and cut transversely, in 1 cm pieces. The rods were then placed in an incubated oven (40 °C) to remove all remaining moisture.

### 2.3. Elucidation of Structural, Thermal and Crystalline Properties of the pH-Responsive Hydrogel

Fourier transform infrared (FTIR) spectra of the non-dried molded rods, dried purified rods and respective starting materials were acquired using a Perkin Elmer Spectrum 2000 FTIR spectrometer, (PerkinElmer Spectrum 100, Llantrisant, Wales, UK) in the range of 650–4000 cm^−1^. Differential Scanning Calorimetry (DSC) analysis was also performed for these materials, employing a Mettler Toledo, Columbus, OH, USA instrument, over an initial run of 25–110 °C, followed by a secondary run of 25–250 °C temperature range, at 10 °C/min for the same sample. XRD was undertaken employing a Miniflex diffractometer (Rigaku, Toyko, Japan) with CuK_α_ (1.5406 Å) radiation. The samples were analyzed over a 0° to 90° 2-Theta range. Results obtained were collated with Rigaku software (Version 2.0), evaluating the degree of crystallinity of the sample.

### 2.4. Gravimetric Analysis of the pH-Responsive Hydrogel

Analysis was carried out via a modified procedure [17,18] where the purified rods (*n*: 3), 1 cm in length, where placed in three respective solvents, (a) deionized water (pH 7.0), (b) simulated intestinal buffer (pH 6.8) and (c) simulated gastric buffer (pH 1.2), all maintained at 37 °C (±0.2 °C) employing an orbital shaker. Simulated gastric buffer comprised of 2 g NaCl, 3.2 g of 1200 u/mg pepsin derived from porcine stomach mucosa, with 7 mL of HCl, made up to 1 L with deionized water, with pH confirmed to 1.2. Simulated intestinal fluid consisted of 6.8 g of monobasic potassium phosphate, 77 mL of 0.2 N NaOH, 1 g of porcine pancreatine, made up to 1 L using deionized water, and pH adjusted to 6.8 using 0.2 N NaOH. 

The initial weight of each purified rod was determined at time zero. At predetermined time intervals (1; 1.5; 2; 2.5; 3 h) the purified rods where weighed accurately and placed back into the respective solvent. Once all the weights of samples were obtained over the time interval, the percentage of swelling was determined according to Equation (1):

SP% = [*Wt_x_* − *Wt*_0_]/*Wt*_0_ × 100, (1)

where, SP% is the swelling percentage, *Wt_x_* is the weight at time *x* and *Wt*_0_ is the weight at time 0.

### 2.5. Physiochemical Properties of the pH-Responsive Hydrogel

The deformation energy (DE) in three different solvents, deionized water, simulated gastric fluid (pH 1.2) and simulated intestinal fluid (pH 6.8) was evaluated. A calibrated textual analyzer (TA.XTplus, Stable Microsystems, Surrey, UK) equipped with a 5 kg load cell, with a cylindrical steel probe of 50 mm diameter for matrix resilience (MR), and a flat tip steel probe of 2 mm diameter for matrix hardness (MH) and DE was utilized. All studies (*n* = 3) were conducted at standard operating procedures (25 °C, 1 atm. Pressure). MR (%) was calculated by the percentage of the ratio between the area under the curve (AUC) of the peak to baseline (after the force is removed; AUC 2–3) and the baseline to peak (before the force is removed; AUC 1–2) from a force-time profile. Matrix hardness is the maximum force required for deformation to occur. MH (N/mm) and DE (J) were both determined based on force-distance profiles. MH was elucidated from the gradient between the initial force and the maximum force attained, whereas DE was calculated from the AUC [19].

### 2.6. Porosity Analysis of the pH-Responsive Hydrogel

Surface area and porosity analysis was performed on the purified rods with respect to time and immersed solvent type using a Porositometric Analyzer (Micrometritics ASAP 2020, Norcross, GA, USA). The in situ purified rods were initially swelled in the respective solvent: water (pH 7.0), simulated intestinal fluid (pH 6.8), simulated gastric fluid (pH 1.2), following freezing with liquid nitrogen at the respective time. The purified rod samples where then lyophilized in order to remove the respective solvent. The purified rod samples where then degassed to aid the removal of surface moisture and contaminants prior to analysis.

Sample preparations briefly involved weighing of each rod and placing them within the sample tube (I.D = 9.53 mm). A glass filler rod was then inserted into the sample tube to reduce the total free space volume within the tube, thus facilitating a reduction in time required for complete degassing to occur. The purified rod samples where completely degassed after a period of approximately 9 h (the process of which encompassed an evacuation and heating phase). Subsequent to complete degassing, the sample tube was transferred to the analysis port where the surface area, pore volume, and pore size of the hydrogel was obtained in accordance with BJH and BET computations. The general standard procedure for the determination of surface area of porous materials is based on the BET gas absorption method, which was conducted in a two-stage process [20]. The BET equation was used in the linear form to determine the monolayer capacity based on Equation (2).

*p*/*n*^a^ (*p*° − *p*) = [1/*n*^a^*_m_* × *c*] + [(*c* − 1) *p*/*n*^a^*_m_ cp*°], (2)

where, *n*^a^ refers to the quantity of N_2_ absorbed at the relative pressure *P*/P_0_. *n*^a^*_m_* was the monolayer capacity and *c* was exponentially related to the enthalpy of absorption in the first absorption layer. The surface area was then determined from the monolayer capacity using Equations (3) and (4). This however, required data regarding the molecular cross-sectional area occupied by the absorbate in the complete monolayer.

*A*_s_ (BET) = *n*^a^*_m_* × *L* × *a_m_*, (3)

*a*_s_ (BET) = *A*_s_(BET)/*m*, (4)

where *A*_s_ (BET) and *a*_s_ (BET) are the total and specific surface areas, respectively, of the adsorbent (of mass *m*). *L* is the Avogadro constant.

### 2.7. Surface Topography of the Swelled Gel

Morphological examination of the surface and pore structure was conducted using scanning electron microscopy (FEI ESEM Quanta 400 F, Hillsboro, OR, USA) at an acceleration of 20.00 kV. The purified rod samples where swelled in the respective solvents (deionized water (pH 7.0), simulated gastric fluid (pH 1.2) and simulated intestinal fluid (pH 6.8)) at predetermined time intervals (0, 60, 90, 120, 150, 180 min). The swelled purified samples where then flash frozen with liquid nitrogen and lyophilized to remove the respective solvent. The dry samples where then imaged using the FEI ESEM Quanta 400 F (FEI™, Hillsboro, OR, USA) electron microscope to obtain high-resolution SEM images, after sputter coating for 90 s. The architectural structures of the swelled purified rods were then compared for differences due to specific solvents employed.

### 2.8. Magnetic Resonance Imaging of the Swelled Gelling Mechanism

The prepared dried rods were placed in a flow through cell employing a benchtop MRI (Maran-i, Oxford Instruments, Oxfordshire, UK), equipped with a 0.5 tesla permanent magnet system stabilized at 37 °C [21,22]. The hydrogel was held in place within a plastic holder using a rubber retaining band. Images were collected over the course of 24 h at predetermined time intervals to visualize the swelling nature of the hydrogel. The hydrogel was allowed to swell within the respective solvent in order to determine the swelling capacity within a continuous flow system at 37 °C. T1-weighted images were captured using a spin echo method and the single intensity of the MRI images were defined according to Equation (5) [22]:

*S* = *M*_0_ × exp − *TE*/*T*_2_ × (1 − exp − *TR*/*T*_1_), (5)

where *M*_0_ is magnetization; *T*_1_ and *T*_2_ are relaxation times; *TR* and *TE* are repetition and echo times respectively.

### 2.9. Rheological Evaluation Undertaken on the pH-Responsive Hydrogel System

The viscosity (η) of the pH-responsive hydrogel was determined at 10, 25 and 40 °C, at a shear rate of 200 s^−1^ using a Modular Advanced Rheometer (ThermoHaake MARS 69 Modular Advanced Rheometer, Thermo Electron, Karlsruche, Germany), equipped with a C35/1° Ti sensor. The viscosity of a 1 mL sample was evaluated over 10,800 s. A solvent trap was utilized during the evaluation to prevent evaporation of the hydrogel sample.

### 2.10. Drug Loading and In Vitro Analysis of the pH-Responsive Hydrogel Employing UPLC Quantification

Drug loading evaluation was undertaken using theophylline as a prototype drug, to demonstrate the efficiency of entrapment of the hydrogel. The bioactive was dissolved in 2% *v*/*v* DMSO/Millipore^®^ water solution, containing a concentration of 0.003 M. The hydrogel was allowed to swell for 24 h, for maximum entrapment, following drying at 40 °C using vacuum oven conditions. UPLC was undertaken for determination of drug loading and release concentration of theophylline (23). UPLC chromatographic separations were performed using an ACQUITY UPLC^®^ High Strength Silica (HSS) T3 column (100 mm × 2.1 mm, 1.8 μm) (Waters, Milford, MA, USA), equipped with array detector (PDA), Empower^®^ Pro software (Waters, Milford, MA, USA), autosampler and 515 dual pumps. The λ was set at 271 nm and the column was maintained at 25 °C. Mobile phase A (0.2% acetic acid) and mobile phase B (acetronitrile) was utilized as a gradient method: 0–10 min, 5–35% B; 10–10.10 min, 35–80% B; 10.10–11 min, 80% B; 11–11.10 min, 80–5% B and 11.10–12.50 min, 5% B at a flow rate of 0.4 mL/min. The area under curve (AUC) for each drug peak, over a wide range of concentrations (25, 20, 15, 10, 5, 2.5, 1.25 and 0.75 mg/L) was determined in order to generate a standard curve for each drug. An internal standard of caffeine (20 mg/L) was utilized throughout the study to validate the accurate retention time and AUC for each procedure. An injection volume of 2 μL was utilized throughout the study.

The estimated percentage of drug entrapment efficiency was determined before dissolution studies were undertaken. A hydrogel, which had been precisely cut into 1 cm long cylinders, crushed into powder form (placed through a sieve of aperture 800 μm) and weighed. The powder was then suspended in 100 mL Millipore^®^ water for 24 h. A 5 mL sample was then withdrawn and filtered through a 0.44 μm Millipore Millex injection filter (Billerica, MA, USA). The filtered sample was placed within an ANSI 2 mL vial and the drug concentration calculated utilizing the previously described UPLC method. This procedure was conducted in triplicate and the entrapment efficiency was determined according to Equation (6).

Drug entrapment efficiency (%) = {Actual drug content/Theoretical drug content} × 100(6)

Drug loaded hydrogel sample of theophylline was placed in a 20 mL glass polytop containing 10 mL gastric simulated fluid for a time period of 24 h. The polytops were incubated within an Orbit Shaker Incubator (LM-530-2, MRC Laboratory Instruments Ltd., Hahistadrut, Holon, Israel) at 37 ± 0.5 °C at 50 rpm. Sink conditions were maintained throughout the study. At predetermined time points, a 1 mL sample was withdrawn and replaced with a temperature-equilibrated aliquot of the respective fluid. The samples were filtered through a 0.22 μm Millipore Millex injection filter (Billerica, MA, USA) and subject to UPLC analysis. The cumulative percentage of drug dissolution and the concentration of drug dissolution were plotted against time. Each drug dissolution study was repeated in triplicate.

## 3. Results and Discussion

### 3.1. Characterization Studies Undertaken on the pH-Responsive Hydrogel

**Figure 1:**
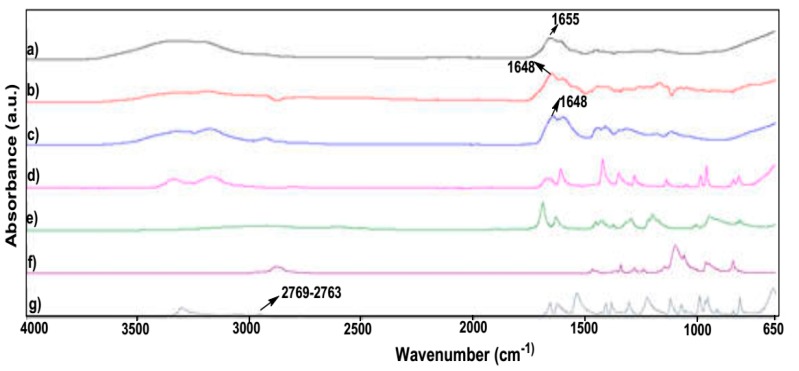
Infrared spectra of (a) synthesized hydrogel; (b) lyophilized hydrogel; (c) polyacrylamide; (d) acrylamide; (e) methylacrylamide; (f) pluronic F-127 and (g) MBA.

A comparative analysis of the respective monomers displayed a common peak between high molecular weight polyamide and the hydrogel at 1648 cm^−1^, due to an amide-I band of polyacrylamide [23,24,25,26] as seen in Figure 1. The peak displayed at 1648 cm^−1^ was due to the high molecular weight polyacrylamide and the hydrogel. Wavenumber peaks present in MBA at 2769 and 1072 cm^−1^ were present in the dried hydrogel at 2763 and 1071 cm^−1^, suggesting the presence of the crosslinker between the monomer units of acrylamide and MAA. A similar peak, which was observed between acrylamide and hydrogel, was present at 1051 and 1049 cm^−1^, respectively. This peak was not observed with polyacrylamide, indicating that the hydrogel displayed –C–O–C– stretching vibrations [27]. FTIR thus confirmed successful free radical polymerization of the hydrogel, possessing substantial polymer swelling properties for stimuli-responsive drug delivery.

**Figure 2:**
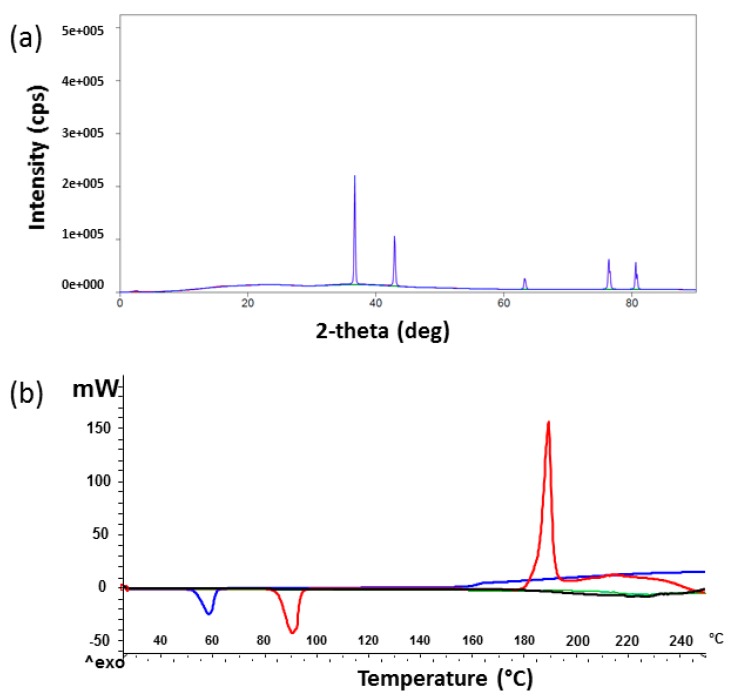
(**a**) XRD evaluation of the hydrogel, reflecting the crystalline nature of the copolymer and (**b**) DSC thermograms reflecting the hydrogel (black line), polyacrylamide (green line), acrylamide (red line) and pluronic F-127 (blue line) at a heating rate of 10 °C between the temperature range of 25–240 °C.

XRD evaluation revealed the crystalline nature of the hydrogel, displaying major reflections at 2θ-values of 36.2°, 42.8°, 63.1°, 77.3° and 80.9°. As seen in Figure 2a, peaks reflected appear narrow in nature, demonstrating clear crystalline elements of the copolymer. The degree of crystallinity was calculated as 65% of the lyophilized copolymer, before reaching aqueous conditions. Differential Scanning Calorimetry of the hydrogel, high molecular weight polyacrylamide, acrylamide and pluronic F-127 was undertaken, as seen in Figure 2b.

The lyophilized hydrogel demonstrated minimum change in thermal characteristics between 25 °C to 185 °C. The lack of thermal transitions observed could possibly be an indication of stability of the hydrogel due to the crosslinking of methylacrylic acid and acrylic acid monomers. High molecular weight polyacrylamide also demonstrated minimal defined thermal events with progressive endothermic migration in the region of ~200 °C. The lack of defined thermal events (melting and crystallization) indicates that the electrostatic bond organization within the polyacrylamide allows for significant resistance to thermal energy input. The acrylamide monomer demonstrated a well-defined melting transition (∆*H*f: −150.89 J/g, onset 84.72 °C, peak 88.74 °C) as well as a crystallization transition (∆*H*c: 380.58 J/g, onset 187.16 °C, peak 197.33 °C). The non-ionic surfactant pluronic F-127 demonstrated a well-defined melting point (∆*H*f: −76.22 J/g, onset 52.56 °C, peak 57.37 °C). An exothermic migration occurred in the region of ~158 °C, suggesting degradation of the polymer.

### 3.2. Gravimetric Analysis of the pH-Responsive Hydrogel

Results obtained from gravimetric swelling analysis of the hydrogel indicate that, within the gastric environment (pH 1.2), the hydrogel exhibited minimum swelling when compared to intestinal environment conditions. The degree of swelling which occurred within simulated gastric media was found to be 193.79%, resulting in a swelling rate of 0.81% min^−1^. This result was significantly low in comparison to swelling of the hydrogel in deionized water and simulated intestinal media. The hydrogel underwent extensive swelling in simulated intestinal environment, approaching ~872% within 4 h, with a swelling rate of 3.63% min^−1^. The swelling potential of this hydrogel within simulated intestinal fluid is ~4 times greater than within the stomach per minute. The hydrogel also underwent extensive swelling within deionized water but was slightly greater than that obtained in simulated intestinal conditions. The degree of swelling within 4 h in deionized water was observed to be 1133.53%, with a swelling rate of 4.72% min^−1^. This indicates that the presence of buffer salts within simulated intestinal fluid restricted the swelling potential of the hydrogel, due to greater concentration of ions in solution, controlling the rate of water absorption. The higher rate of swelling observed in deionized water with respect to simulated intestinal buffer was simply due to different osmotic concentration of the two media. Gravimetric analysis thus confirmed that the hydrogel is highly responsive to pH, indicating its suitable application for targeted drug delivery in the GIT. With respect to recently reported hydrogels within this class of “pH responsive hydrogels possessing macroporous interiors resembling a honey-comb framework”, Suhag and co-workers, 2015, reported copolymerization of acrylic acid (AAc) and 2-(dimethylamino)ethyl methacrylate (DMAEMA) to form poly(AAc–*co*–DMAEMA) (PAD) hydrogels. Although the best hydrogel formulation PAD90 showed equilibrium-swelling ratio (ESR) of 4027% at pH 7.0, the rate of swelling was very slow with 121% swelling over a period of 30 h. It was further observed that the hydrogel with a lower ESR of 2296% demonstrated 60% swelling within the same period (1391%) [28].

### 3.3. Mechanism of Swelling of the SPH

**Figure 3:**
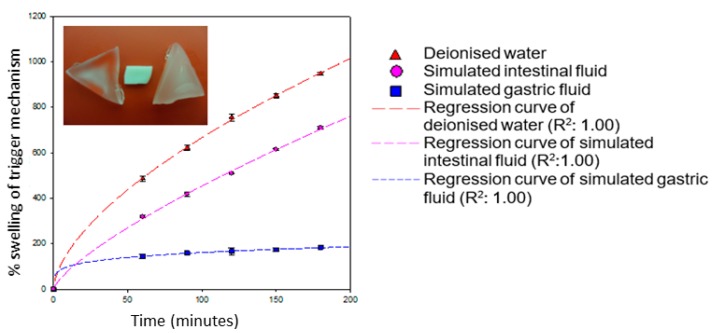
Swelling studies of the pH-responsive hydrogel in the respective solutions of deionized water (pH 7.0); simulated gastric fluid (pH 1.2) and simulated intestinal fluid (pH 6.8), with respect to percentage of swelling and time in minutes. Regression curves where fitted onto each respective scatter plot with an *R*^2^ value of 1.00.

The swelling mechanism of a SPH is dependent on the functional groups that exist within the crosslinked polymers. A typical example of this is the presence of –COOH functional groups that undergo ionization at higher pH values and are protonated at lower pH. The ionization of –COOH induces the formation of an increased concentration of counter ions within the network structure of the SPH [29]. In response to the increased concentration of counter ions, osmotic pressure within the network is equilibrated by the movement of water molecules into the network structure, thus induction of swelling commences. The greater the osmotic pressure that can be generated within the network of a hydrogel, the greater the extent of network swelling that may occur. As seen in Figure 3, results indicate that with an increase in pH from acidic to basic, a considerable increase in swelling was observed for the hydrogel within a relatively short period of time (4 h). The presence of methacrylic acid increases with swelling of the superporous hydrogel in basic solutions and collapses in acidic solutions [6,29]. This increase in swelling ratio was due to the dissociation of carboxyl groups of methacrylic acid, thereby increasing the osmotic pressure inside the hydrogels, resulting in increased swelling. At high pH values, the carboxylate side chains are repelled by the ions in the solution and minimize the charge concentration by expanding. Swelling mainly depends upon the extent of crosslinking and the type of crosslinker with respect to the monomer concentration within the hydrogel network [30,31].

The swelling potential of the hydrogel is minimized within the acidic conditions of the simulated gastric environment. This is due to the protonation of carboxylate functional groups within methylacrylic acid. The protonation of the carboxylate functional groups causes the formation of negative charged sites within the SPH network. Once the negative charge is stabilized within the network of the SPH, the collective swelling of the SPH is reduced.

### 3.4. Physicomechanical Analysis of the pH-Responsive Hydrogel

The simulated gastric compression stress profiles indicate that as the dynamic swelling hydrogel is exposed to simulated gastric fluid over a period of 24 h, the matrix resilience increases. In addition, the weight of the hydrogel also increases with respect to time, with an increase in weight of ~100% from 1 to 24 h. Thus, the increase of matrix resilience within simulated gastric fluid is primarily due to the ordering of hydrogen bonding within the internal structure of the hydrogel within proximately close functional groups. The ability of the hydrogel to withstand a compressive force was greatest with the first 40% strain compression, followed by the 50% strain compression and finally the 40% strain compression repeat. It should be noted that the difference between the matrix resilience between these significant strain compressions was minimal (±4%).

**Figure 4:**
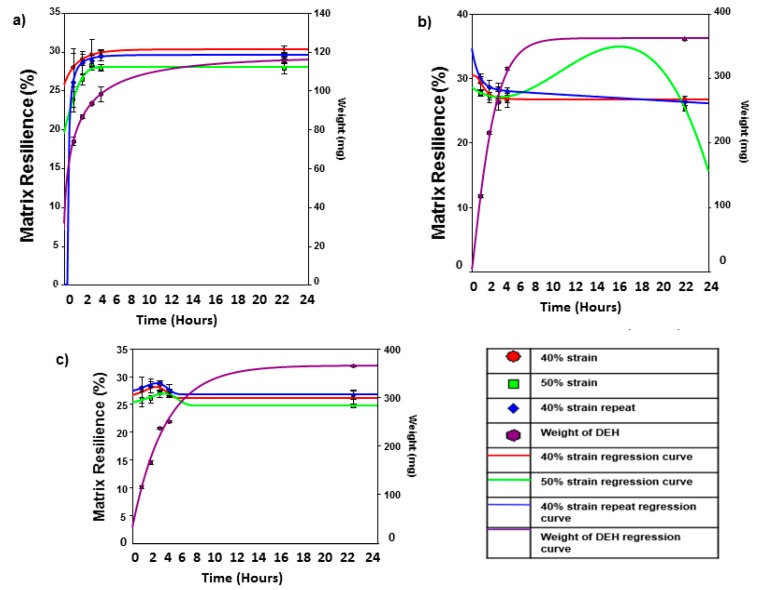
Scatter plots of the hydrogel in simulated gastric buffer (**Graph a**); simulated intestinal buffer (**Graph b**) and deionized water (**Graph c**).

As seen in Figure 4, the simulated intestinal compression stress profiles indicate that the hydrogel generally decreased in matrix resilience when exposed to simulated intestinal fluid over a period of 24 h. This is probably due to the presence of metal ions, which occupy functional groups within the hydrogel structure, thus neutralizing intermolecular bonds between these groups. The weight increase of the hydrogel was also included within the scatter plot, indicating an increase of weight of the hydrogel to ~400% from 1 to 24 h. The swelling rate is relatively similar to that obtained with gravimetric analysis (~4 times greater than that obtained within simulated gastric fluids with respect to simulated intestinal fluids). The deionized water compression stress profiles indicate that the hydrogel shared aspects from both the simulated gastric and intestinal fluids when exposed to deionized water over a period of 24 h. Initially, the hydrogel increased in matrix resilience with respect to time over the first ~180 min, following a decrease after this time period. This was due to the initial formulation of directional hydrogen bonding within the hydrogel structure by the presence of deionized water. Once the concentration of deionized water within the hydrogel achieved a critical level, the intermolecular hydrogen bonding between proximately close function groups failed to be maintained. Thus, directional intermolecular hydrogen bonds were replaced with water cages around the functional groups, thus allowing bulk water to move through the structure that did not reinforce the matrix resilience of the hydrogel. This is evident by the absence of salt ions (as in simulated intestinal buffer), which could have disrupted the intermolecular hydrogen bonding, resulting in a reduction in matrix resilience, similar to that within simulated intestinal buffer.

### 3.5. Porosity, Morphological and Megnetic Resonance Imaging Evaluation of the pH-Responsive Hydrogel

**Figure 5:**
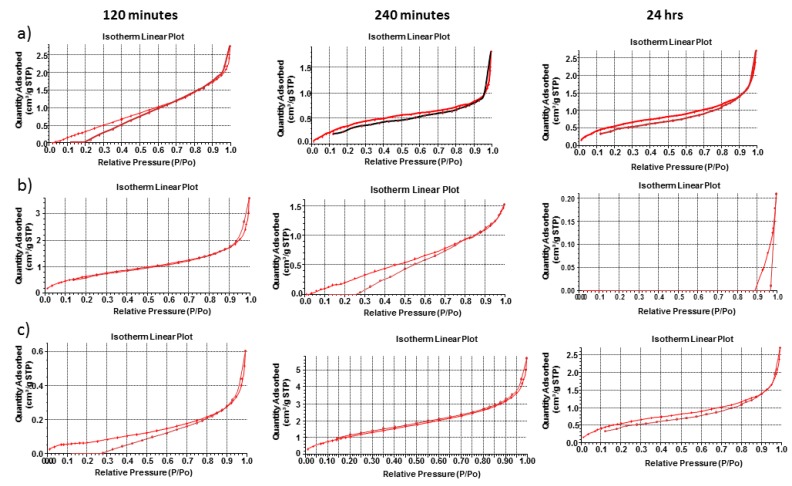
Linear plot isotherms of the hydrogel over 24 h in (**a**) intestinal simulated medium (pH 6.8); (**b**) gastric simulated medium (pH 1.2) and (**c**) deionized water (pH 7).

**Figure 6:**
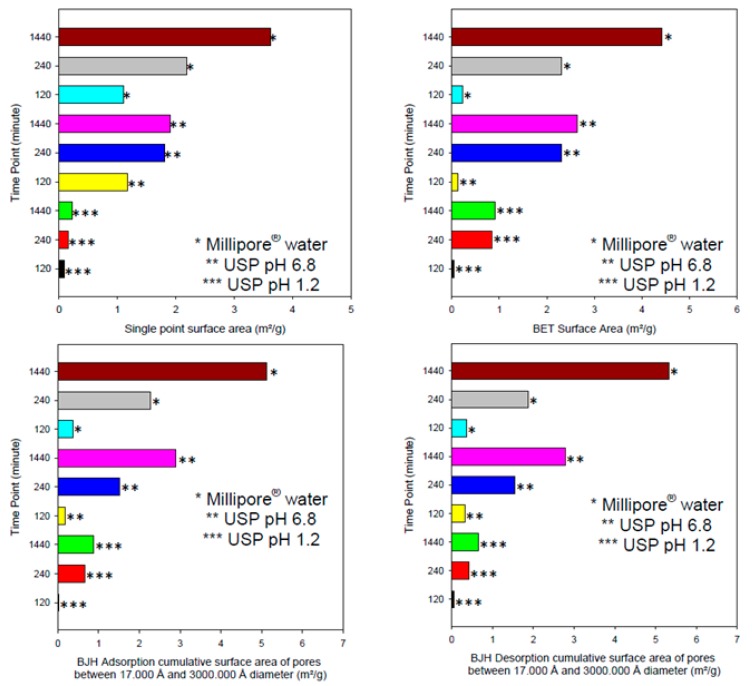
Porositometric surface area analysis with respect to time of the hydrogel within simulated gastric fluid (pH 1.2), simulated intestinal fluid (pH 6.8) and Millipore^®^ water.

The hydrogel within simulated gastric fluid demonstrated a very small increase in single point surface area (increase of ~0.4 m^2^/g), as seen in Figure 5 and Figure 6. In addition, the BET surface area increases but BJH absorption and desorption cumulative surface area of pores between 17 and 3000 Å decreases with respect to time. This suggests that the pores that fell within this range decreased in number as the hydrogel was exposed to simulated gastric fluids. This is evident by the increase in pore size (as seen in the adsorption average pore width and the BJH absorption/desorption average pore diameter increase). It was observed that the pore volume decreased as the hydrogel was exposed within simulated gastric fluids. The dynamic change in pore volume could be due to movement of simulated gastric fluid into the hydrogel structure and the formation of new intermolecular bonds that induce microscopic changes in pore volume as new bonds were equilibrating with respect to time. The dynamic change in pore volume could be achieved as small pores expanded (due to movement of gastric fluids into the hydrogel) outside the measured range of 17 to 3000 Å diameter pores.

**Figure 7:**
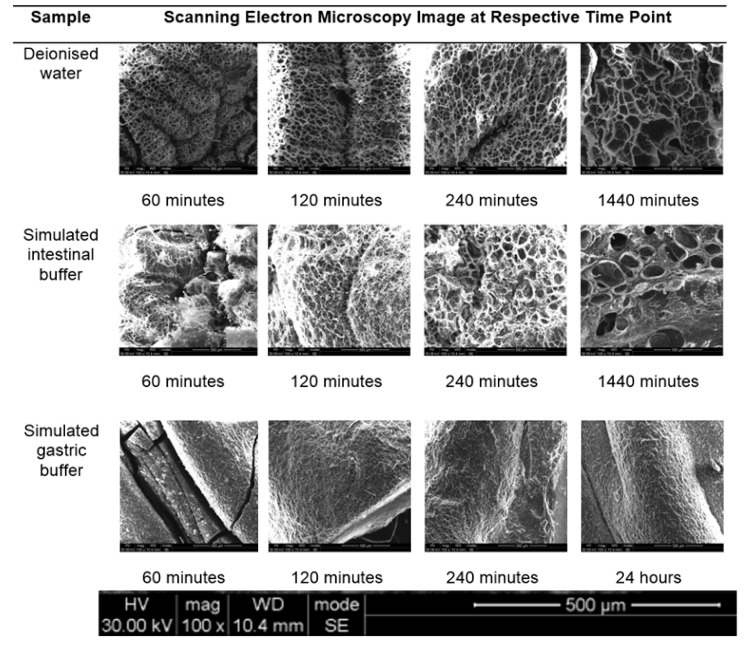
SEM images of the hydrogel within deionized water, simulated intestinal fluid and simulated gastric fluid at predetermined time points over a period of 24 h. All samples were analyzed at 100 times magnification with the scale bar of all samples represented in the bottom of the figure.

The hydrogel within simulated intestinal fluid was difficult to measure due to the presence of salt ions within the structure of the hydrogel. The hydrogel initially demonstrated an excellent analysis of surface area, pore volume and pore size (all of which were significantly larger than the hydrogel within simulated intestinal fluid within the same time period). The salt ions within the simulated intestinal fluid could have also disrupted the pore structure, as salt crystals would have formed within the pore structures during the freeze-drying process. An average BET adsorption pore size in gastric and intestinal medium produced pores in the range of 17,000 and 3,000,000 Å respectively. These results were closely correlated to SEM pore size evaluation, of which gastric medium produced pores in single digit microns, and intestinal pH resulting in pore sizes of 280–320 µm, as seen in Figure 7. BET adsorption and SEM morphological pore size evaluation thus correlated significantly with respect to pH variation. SEM studies clearly demonstrated a non-porous arrangement of the polymer in gastric medium, with contrasting highly porous network structures in intestinal medium.

The hydrogel within deionized water demonstrated significant increases in single point surface area (increase of ~3.4 m^2^/g). This was also true for the BET surface area which increased by ~4.2 m^2^/g. According to the results obtained from the porosity analysis of the hydrogel, simulated gastric fluids generated the smallest change in both pore size and surface area with respect to simulated intestinal fluids and deionized water. This can be explained due to the functional groups present within the hydrogel chemical structure, becoming ionized in basic pH conditions, resulting in substantial swelling of the hydrogel polymeric matrix. The hydrogel thus displayed an H1-hysteresis loop, with a type IV isotherm in simulated gastric fluid and deionized water. SEM images obtained of the hydrogel within the 3 mediums, as seen in Figure 7, correlate closely with gravimetric swelling analysis.

As seen in Figure 7, the pore size of the hydrogel within deionized water was larger and more uniform in comparison to the pore structure within simulated intestinal fluid. Additionally, the hydrogel within deionized water underwent significantly less alteration in pore morphology, due to the absence of the metal ions. The wall thickness between the pores of the hydrogel in simulated intestinal fluid was far greater than that of deionized water, indicating that maximum swelling of the hydrogel was retarded due to the presence of metal ions. The hydrogel within simulated gastric fluid remained homogenous with little to no observation of a porous network structure. Since the hydrogel within simulated gastric fluid was highly charged, this contributed to the retarded development of a porous network structure [32,33,34,35].

**Figure 8:**
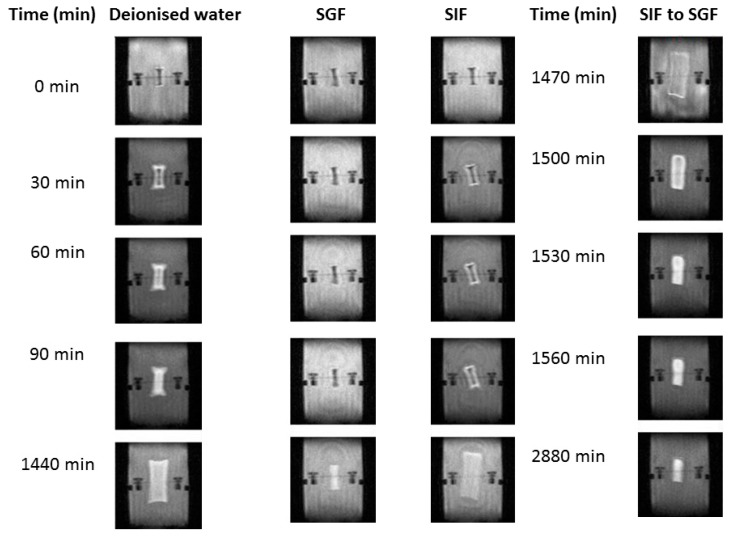
pH-responsive evaluation of the hydrogel mechanism was evaluated using MRI within deionized water, simulated gastric fluid (SGF) (pH 1.2) and simulated intestinal fluid (SIF) (pH 6.8). After 1440 min of evaluation of the sample in SIF, the same sample was introduced to SGF with images obtained up to 2880 min.

The utilization of MRI technology was used to validate the ability of swelling of the hydrogel with respect to simulated gastric and intestinal fluids, as well as identification of degradation of the hydrogel, evaluated using MRI imaging. The hydrogel dosage form was designed as a maximum 24 h oral dosage formulation; therefore evaluation greater than 24 h in the respective medium was not necessary. Over 24 h, the hydrogel continued to display images of increasing swelling, rather than images demonstrating degrading polymer mass, thus indicating a positively functional platform over 24 h. In simulated gastric buffer (pH 1.2), the hydrogel displayed minimum swelling over 24 h, as seen in Figure 8. On the contrary, it was observed that the hydrogel demonstrated exponential swelling in simulated intestinal fluid and deionized water, maintaining a relatively continual swelling profile over 24 h. Additionally, images demonstrated that the hydrogel did not degrade rapidly within simulated gastric fluid (images demonstrating intact surface outlines of the hydrogel platform), confirming the intact structure once reaching the small intestine where rapid swelling and release of its loaded contents will occur, as proved in Figure 8. Once again, when evaluating the hydrogel in intestinal medium, it was observed that the hydrogel was able to swell rapidly but not as rapidly as in deionized water, due to osmotic concentration differences in the respective medium. The ability of the hydrogel to undergo reversible swelling when the environment was changed from neutral to acidic, further demonstrates the dynamic ability of the hydrogel to be pH-stimuli responsive. Thus, the hydrogel demonstrated that a change in pH induces significant swelling abilities, even when interconversion of pH medium is carried out.

### 3.6. Rheological Evaluation of the pH-Responsive Hydrogel 

**Figure 9:**
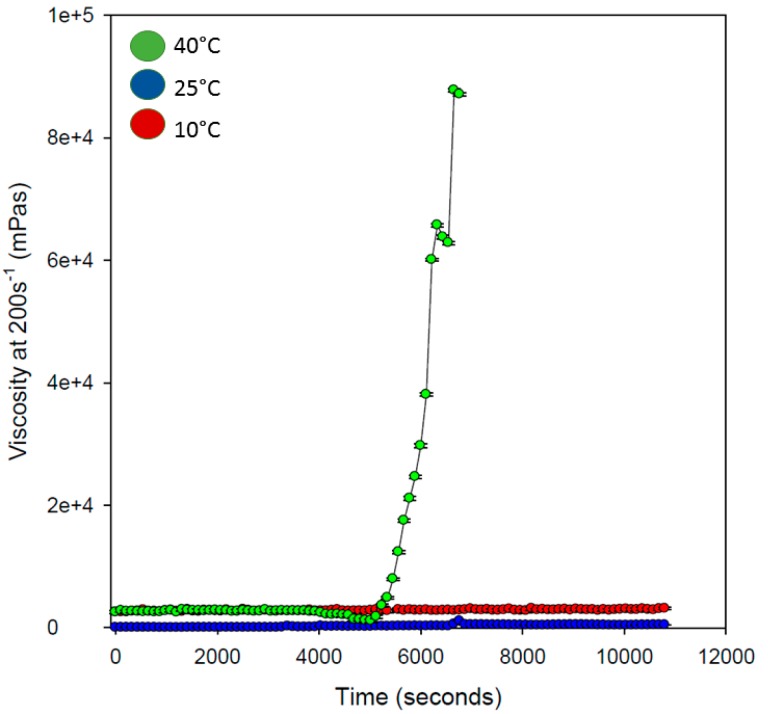
Rheological evaluation of the hydrogel at 10, 25 and 40 °C, for determination of change in viscous nature of the pH-responsive hydrogel.

Rheological analysis of the hydrogel was determined with variations in temperature, to determine the effect on viscous nature of the hydrogel with respect to a change in temperature. As seen in Figure 9, the hydrogel remained uniform in its viscosity over the entire duration of analysis at 10 and 25 °C. However, at 40 °C, the viscous nature of the hydrogel changed after approximately 4500 s (75 min), increasing exponentially which may be due to gelification of the hydrogel copolymeric network (transition sol/gel). It was observed that the hydrogel increased in viscosity approximately 90,000 times at this temperature, after 7000 s (117 min), further exemplifying the ability to load drug in the copolymeric hydrogel network. As discussed in the following section, drug loading was evaluated as >98%, possibly due to one of the advantages of superior exponential viscous transition experienced in the copolymeric hydrogel system. This property of the hydrogel would also promote increased mucous adhesion in the small intestine, further promoting the release of loaded drug at the site of absorption.

### 3.7. Drug Loading and In Vitro Evaluation of the pH-Responsive Hydrogel Employing UPLC Quantification

**Figure 10:**
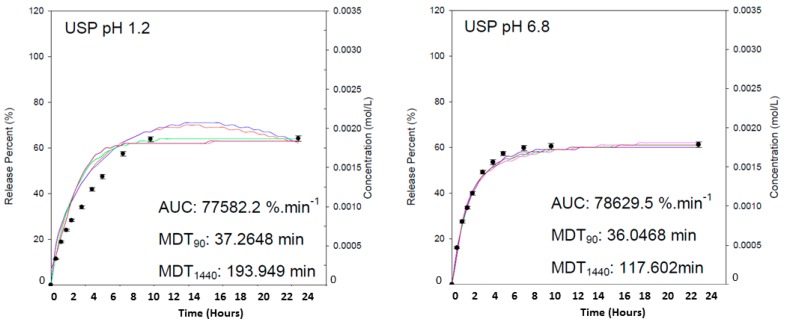
Drug release profiles using theophylline as a prototype drug, evaluating the release kinetics of the hydrogel over 24 h in gastric and intestinal simulated fluids.

Drug entrapment efficiency of the hydrogel employing theophylline (0.003 M) was calculated as 98.2%. The substantial effect of the copolymer to absorb an extensive percentage of drug can be correlated closely to the gravimetric swelling analysis of the hydrogel, where swelling within 4 h in deionized water was observed to be 1133.53%. The AUC was employed for determination of drug concentration, indicating the extent of drug release. As seen in Figure 10, greater amount of drug was released in intestinal simulated fluid over a 24 h duration, in comparison to the release kinetics observed in gastric simulated fluid.

It was observed that maximum release of the drug was obtained within 10 h of release analysis in both pH conditions, demonstrating the controlled nature of swelling potential of the dynamic hydrogel formulation. It was observed that 19% of drug was released in 90 min, accounting for gastric emptying, and potentially possessing 45% of the remaining drug to be release once reaching the small intestines. It can be explained that the presence of –COOH functionalities in the copolymer, undergoing ionization at higher pH values induces the formation of an increased concentration of counter ions within the network structure [30]. In response to the increased concentration of counter ions, osmotic pressure within the network is equilibrated by the movement of water into the copolymer matrix, thus the extent of swelling occurs. The greater the osmotic pressure that can be generated within the network of a hydrogel, the greater the extent of network swelling that may occur. This platform can thus be applicable for sensitive bioactives due to the minimum concentration of release in gastric environments within gastric emptying duration, allowing maximum loaded drug to be released and absorbed upon reaching the small intestines.

### 3.8. Performance Analysis of the pH-Responsive Hydrogel

The materials used in the formulation pH responsive hydrogel were composed of acrylamide, MAA and Pluronic F127. To the best of our knowledge, in terms of composition, no such interpenetrating polymer network (IPN) superporous hydrogel has been reported before. Ni and co-workers (2007) prepared polyacrylamide-methacrylic acid hydrogel microspheres and reported a sharp pH–volume transition in the magnitude of approximately 12 times with an increase in pH by 0.5. However, the hydrogels showed a decrease in swelling after a maximum pH was reached (which was still below intestinal pH) probably due to degradation of scattering domains [36]. Gupta and Shivakumar (2012) also synthesized superporous hydrogel containing poly(methacrylic acid-*co*-acrylamide) and concluded that these hydrogels can be employed for pH-responsive drug delivery [37]. More recently, Baskan and co-workers (2013) reported poly(acrylic acid)/Pluronic IPN hydrogels and discovered the matrix toughening and stabilization properties of Pluronic in the developed hydrogels [38]. The Aam/MAA copolymeric hydrogel developed in this research provided a unique hydrogel with extensive swelling capacity of 872% (after a duration of 4 h) in intestinal simulated fluid at approximately 3.63% min^−1^. The synthesized hydrogel also possesses significant drug loading capacity >98%, with maximum drug release within 10 h. Given the upper GIT emptying time of ≈8–12 h, a rapid swelling kinetics is of ~872% within 4 h shown by the hydrogel reported in this report will be most beneficial for time-efficient delivery of bioactives orally [39]. The hydrogel reported in this report demonstrated an optimal equilibrium swelling with high swelling rate. Most importantly, the hydrogel displayed matrix retention over the GIT emptying time, which may be due to the presence of Pluronic F127 in the matrix. Such matrix hydrogels may provide a cost-effective alternative to drug delivery systems with pH-responsive coating wherein uneven coating and dose dumping can be avoided.

## 4. Conclusions

A pH-responsive hydrogel system was prepared by free radical polymerization of acrylamide and methacrylic acid in the presence of *N*-*N*′-Methylenebis(acrylamide). The hydrogel was designed as a versatile oral polymeric delivery platform, with the objective to encapsulate and protect gastric-sensitive bioactives, following substantial swelling >870% upon reaching intestinal pH, thereby successfully delivery the encapsulated bioactive at the site of absorption. The hydrogel further demonstrated superporous network architecture, ideally designed for substantial water absorption in intestinal pH conditions. Chemical and thermal evaluation employing FTIR and DSC respectively, further confirmed crosslinking following free radical polymerization. Morphological analysis using SEM and MRI, confirmed the porous and swelling capacity of the hydrogel system, in response to gastric and intestinal pH conditions. Drug loading and release evaluation confirmed >98% drug loading capacity, with over 60% drug release in 24 h, due to a swelling capacity of approximately 4.72% min^−1^. It can thus be concluded that the hydrogel oral drug delivery system has significant potential as a site-specific pH-responsive copolymeric platform, with the objective to potentially administer gastric-sensitive therapeutics via the oral route of delivery.
